# Humidity-Sensing Chipless RFID Tag with Enhanced Sensitivity Using an Interdigital Capacitor Structure

**DOI:** 10.3390/s21196550

**Published:** 2021-09-30

**Authors:** Junho Yeo, Jong-Ig Lee, Younghwan Kwon

**Affiliations:** 1 School of ICT Convergence, Daegu University, 201 Daegudae-ro, Gyeongsan 38453, Gyeongbuk, Korea; 2 Department of Applied Electronics Engineering, Dongseo University, Busan 47011, Korea; leeji@gdsu.dongseo.ac.kr; 3 Department of Chemical Engineering, Daegu University, 201 Daegudae-ro, Gyeongsan 38453, Gyeongbuk, Korea; y_kwon@daegu.ac.kr

**Keywords:** chipless radio frequency identification (RFID), interdigital capacitor (IDC) structure, humidity sensing, polyvinyl alcohol (PVA), radar cross-section (RCS)

## Abstract

An eight-bit chipless radio frequency identification tag providing humidity sensing and identification information is proposed. A compact, enhanced-sensitivity resonator based on an interdigital capacitor (IDC) structure is designed for humidity sensing, whereas seven electric-field-coupled inductor capacitor (ELC) resonators are used for identification information. These eight resonators are placed in a two-by-four array arrangement. A step-by-step investigation for the effect of varying the number of elements and array configuration on the resonant frequency and radar cross-section (RCS) magnitude of the IDC resonator is conducted. The RCS value of the resonant peak frequency for the IDC resonator increases as the number of array elements placed nearby increases due to the mutual coupling among the elements, and the increase in the RCS value becomes larger as the number of arrays increases in the vertical direction. Polyvinyl alcohol (PVA) is coated on the IDC-based resonator at a thickness of 0.02 mm. A non-reflective temperature and humidity chamber is fabricated using Styrofoam, and the relative humidity (RH) is varied from 50% to 80% in 10% intervals at 25 °C in order to measure a bistatic RCS of the proposed tag. The humidity sensing performance of the IDC resonator in the proposed tag is measured by the shift in the resonant peak frequency and the RCS value, and is compared with a single ELC resonator. Experiment results show that when RH increased from 50% to 80%, the sensitivities of both the resonant peak frequency and the RCS value of the IDC resonator were better than those of the ELC resonator. The variation in the RCS value is much larger compared to the resonant peak frequency for both IDC and ELC resonators. In addition, the resonant peak frequency and RCS value of the PVA-coated IDC-based resonator change, whereas those of the other seven resonators without a PVA coating do not change.

## 1. Introduction

Radio frequency identification (RFID) technology uses electromagnetic waves in various radio frequency bands and identification (ID) information transmitted from tags attached to objects or people in order to automatically recognize the tag-attached object in a noncontact manner [1]. It was developed and used as an early-stage technology in the Internet of Things (IoT), which is a core technology of the fourth industrial revolution. It can be considered a next-generation automatic recognition technology to secure visibility through product tracking and history management by supplementing the shortcomings of barcodes used in logistics and supply chain management. It has been widely used in real life, such as transportation cards, highway toll payments, for clothing and book theft prevention, parking management, access control, food waste management, e-passports, etc. [2,3,4]. The RFID system consists of a tag attached to an object to provide identification information, and a reader that processes information by communicating with the tag. 

RFID tags are classified as chipped or chipless, depending on the presence of semiconductor-based integrated circuits [5]. Chipped RFID tags use a semiconductor process to make memory and the main circuits, whereas chipless RFID tags do not use semiconductors. Chipped RFID tags can store a variety of information, and have the advantage of a miniaturized tag, but it is difficult to lower the price of a chip manufactured by a semiconductor process. Chipless RFID tags can be manufactured at low cost because they do not use a chip, but there are limitations on performance, such as recognition distance and memory storage. Chipless RFID tags can also be classified according to the operational method, such as magnetic material-based, printed electronic circuit-based, and microwave resonator-based [6].

Depending on the electromagnetic waves reflected by the tag, the microwave resonator-based method for chipless RFID tags can use a time domain (or temporal) method, a frequency (or spectral) domain method, or a hybrid method [7,8]. In the time domain method, a delayed transmission line including discontinuous reflectors or complex impedances is configured at a specific position on the substrate of a tag, and the identification code is created by the reflections from the tag. In order to avoid overlapping reflected pulses, and to generate a measurable delay, a transmission line must be implemented with a long length, or a very narrow pulse must be used, so the information density per surface is low, and thus, the bit-encoding capability is limited. The frequency domain method is implemented with multiple resonators adjusted to different predefined frequencies in a specific frequency band included in the interrogation waves from the reader. The method determines the absence or presence of singularities in the amplitude and/or phase of the frequency response of the tag. Therefore, the interrogation waves from the reader must have sufficient frequency bandwidth to cover all the resonant frequencies of the resonators in the tag. It can be classified as retransmission-based or backscattering-based, according to the interaction method between the interrogation wave and the tag. The retransmission-based tag is equipped with cross-polarized transmitting and receiving antennas to communicate wirelessly with the reader, and these antennas are used to receive an interrogation wave from the reader and transmit returned waves from the tag. In the backscattering-based tag, the resonators provide spectrum signals through singular points in the response of a radar cross-section (RCS), and the tag size can be reduced because the tag does not require use of transmit and receive antennas. The hybrid method creates information simultaneously in one or more domains, such as frequency phase, frequency amplitude, frequency bandwidth, polarization diversity, etc., in order to increase the information density per tag substrate surface.

There have been many attempts to incorporate sensing functions into RFID tags [9,10,11]. By combining the sensing function with existing RFID technology, environmental information such as temperature and humidity can be obtained remotely, along with ID information. Such RFID sensors can be used to implement low-cost, identifiable sensors to realize the IoT in real life. For a conventional chipped RFID, an ultra-high frequency (UHF) RFID tag chip (with an integrated temperature sensor and a sensor interface with an analog-to-digital converter) is available on the market and is used as a temperature sensor for concrete maturation monitoring [12]. A probe-augmented T-match dipole was designed in order to place the tag chip 15 cm deep into the concrete. However, the addition of an integrated sensor and the sensor interface increases the cost of the tag chip. To reduce the tag’s cost, a tag antenna or an impedance matching network is used as a sensor to detect variations in environment parameters, such as temperature, humidity, or strain. A temperature sensor tag for passive UHF RFID systems was proposed by using distilled water as a temperature-sensitive material [13]. A water pocket is placed close to the meander-line impedance-matching network situated between the tag antenna and the IC. It turned out that the frequency of the lowest power-on tag increased linearly as temperature increased from 8 °C to 92 °C. Poly(3,4-ethylenedioxythiophene): poly(styrene sulfonic acid) (PEDOT: PSS) was deposited within the glass-like slot of a UHF RFID tag antenna for wirelessly sensing humidity [14]. The minimum turn-on power for the tag increased as humidity increased from 50% to 100%. The wider the area covered by PEDOT:PSS in the slot, the larger the variation in turn-on power. A passive RFID strain sensor using a stretchable meander-line dipole antenna was introduced [15]. The input impedance, gain, power transmission coefficient, and backscattered power of the tag were simulated under stress with respect to strain. The real and imaginary parts of input impedance varied significantly as strain increased. Below the yield point, where strain was about 6%, the tag recovered its original shape when the applied stress was removed, and the backscattered power increased monotonically. However, once the strain exceeds the yield point, deformation becomes permanent and non-reversible, and the backscattered power decreases gradually.

In recent years, many researchers have studied wireless sensors based on chipless RFID technologies using integrated sensors or smart sensing materials. The integration of the sensing material into a chipless RFID tag can be classified into two methods. The first is coating the sensing material over the existing chipless RFID tag as a superstrate, whereas the second is use of the sensing material as a substrate [16]. 

For temperature sensing, commercial semiconductor temperature sensors were integrated with chipless RFID tags, or temperature-dependent sensing materials were used to change the response of chipless RFID tags. An ultra-wide band (UWB) surface acoustic wave (SAW) chipless RFID tag using a spread spectrum approach based on orthogonal frequency coding and the temperature dependence of the SAW velocity for a piezoelectric YZ lithium niobate substrate was proposed [17]. It was tested in the temperature range between 10 °C and 190 °C at 5 °C increments. A chipless RFID tag based on multiple cascaded spiral slot resonators with an integrated thermistor temperature sensor was designed [18]. The resistance of the thermistor decreased nonlinearly when temperature increased from 0 °C to 100 °C. The input reflection coefficient of the monopole tag antenna decreased with an increase in temperature. A passive, wireless temperature sensor based on delay lines and reflectors using barium–strontium–titanate (BST) ceramic as a temperature-sensing material was introduced [19]. The ID-modulating part and the sensing part were fabricated separately, and are connected by a monopole antenna. Permittivity and associated capacitance of the BST decreases as temperature increases. A time-coded UWB chipless RFID sensor tag was proposed, consisting of a UWB antenna connected with a delay line loaded with a positive resistive temperature sensor [20]. The tag mode amplitude of the input reflection coefficient increased when temperature increased from 30 °C to 130 °C. Another chipless RFID temperature sensor uses three spiral resonators coupled with a microstrip line and a temperature-dependent Stanyl TE200F6 polyamide polymer material. The largest spiral resonator was modified as a temperature sensor by means of placing the polyamide between the arms of the spiral and one part of the substrate. The permittivity of the polyamide increased from 3.7 to 4.2 as the temperature increased from 0 °C to 50 °C. Therefore, the resonant frequency of the largest spiral decreased when the temperature increased.

For humidity sensing, various sensing materials, such as ceramics, polymers, and hybrids of ceramics and polymers, have been widely used [21]. Ceramics are inorganic, non-metallic, solid materials made up of either metal or non-metal compounds that have been shaped and then hardened at high temperatures. Polymers are organic macromolecules composed of repeated unit structures, and most of them are carbon–hydride compounds or their derivatives [22]. The functional groups, along with the basic structure of the backbone, determine the chemical and physical properties of the polymers. A group delay-based chipless RFID humidity sensor tag was presented that uses a cascaded group of transmission-line sections (C-sections) and silicon nanowires (SiNWs) deposited on the strips of the C-section group [23]. A group delay change of about 22.3 ns and an RCS decrease of 30 dB were measured near the fundamental frequency at 3.7 GHz when humidity varied from 60.2% to 88%. Relative permittivity and the loss tangent of silicon nanowires increased with water absorption caused by a humidity increase. A compact chipless RFID tag with both identification and sensing capabilities was presented using SiNWs and five C-like strip resonators. The SiNWs were deposited on the largest resonator, and variation of the RCS was measured when both temperature and humidity changed [24]. The peak resonant frequency of the RCS shifted towards a higher frequency when humidity decreased from 75% at 3 °C to 43% at 19 °C. A similar attempt was tried for a group of eight coupled loop resonators using SiNWs [25]. In this case, the SiNWs were deposited vertically in the middle of the resonators. For a fixed temperature of 23 °C, the peak resonant frequency and the magnitude of the RCS decreased when humidity increased from 74% to 98%. A passive, wireless sensor for simultaneous remote sensing was introduced, consisting of a spiral inductor and an interdigital capacitor (IDC) fabricated on a silicon wafer using graphene oxide (GO) as a sensing material [26]. The resonant frequency and maximum real part of the input impedance decreased as humidity increased at fixed temperatures, whereas the same trend was observed when the temperature increased at a fixed humidity. A depolarizing chipless RFID humidity-sensing tag was proposed, using three sets of nested concentric square rings with chamfering on the main diagonal and splits on the vice diagonal, and polyvinyl alcohol (PVA) film deposited on the inner split square ring. Its sensing performance was simulated by changing the relative permittivity of the PVA [27], and the resonant frequency moved towards a lower frequency when the relative permittivity of the PVA increased. A chipless RFID humidity sensor using an electric-field-coupled inductor capacitor (ELC) resonator and PVA was proposed for a microwave frequency band [28]. Two horn antennas were used to measure the variation on the transmission coefficient when humidity varied from 35% to 85%. The resonant frequency of the transmission coefficient shifted towards a lower frequency with a maximum frequency shift of 270 MHz, and the magnitude of the resonant frequency increased. A chipless RFID humidity sensor based on a finite three-by-three artificial impedance surface (AIS), consisting of three square loops per unit cell, was proposed [29]. The AIS was printed on a thin polyethylene terephthalate (PET) paper coated with PVA and aluminum oxide, which as used as a humidity sensing material. The resonant peak frequencies of the AIS shifted towards a lower frequency when humidity increased from 50% to 90%. Recently, the humidity-sensing performance of PVA was compared with other hydrophilic polymers, poly (methyl methacrylate) (PMMA) and poly (2-hydroxyethyl methacrylate) (PHEMA), using a microwave sensor based on a modified interdigital capacitor-shaped defected ground structure in a microstrip transmission line [30]. It was found that the performance of the PVA-coated microwave sensor was the best among the three polymers when sensing performance was measured by the shift in the resonant frequency and magnitude level of the transmission coefficient when relative humidity varied from 40% to 90% at a temperature of 25 °C.

In this paper, an eight-bit chipless RFID tag with identification information and a humidity sensing capability is proposed. First, a compact, enhanced-sensitivity resonator based on an interdigital capacitor (IDC) structure is designed. The IDC structure is employed to enhance sensitivity to variations in permittivity when PVA is coated on its surface, compared to the existing ELC resonator [28], and to miniaturize the size of the ELC resonator, which is used as a high-sensitivity resonator among frequency domain chipless RFID tags. Next, an eight-bit chipless RFID tag in a two-by-four array consisting of one IDC compact resonator and seven ELC resonators was designed. The IDC resonator is used for humidity sensing, whereas the seven ELC resonators are used for identification information. The effects of varying the number of elements and array configuration on the resonant frequency and RCS magnitude of the IDC resonator are systematically investigated. All the simulated results in this paper were obtained using CST Studio Suite (Dassault Systèmes Co., Vélizy-Villacoublay, France) [31].

## 2. Design of Compact Resonator with Enhanced Sensitivity Using IDC Structure

**Figure 1:**
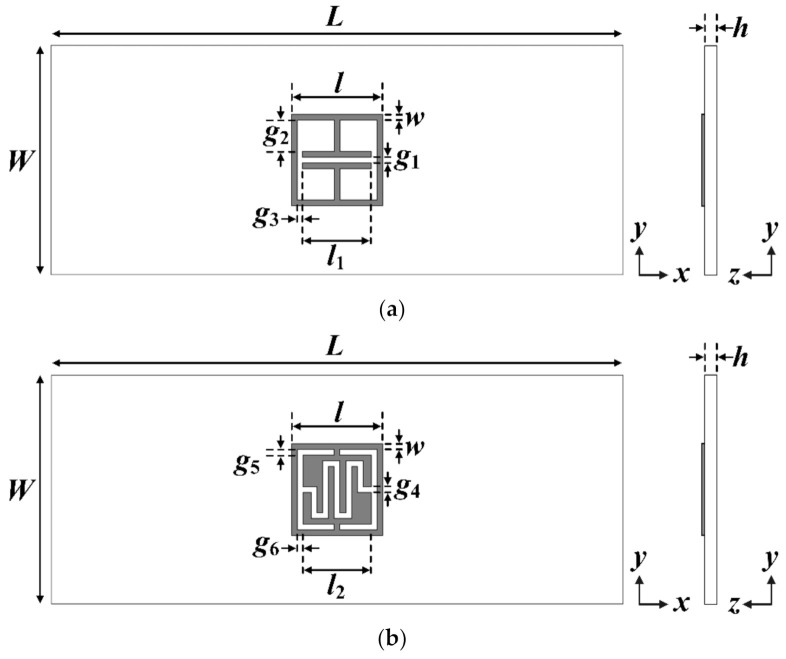
Geometries of (**a**) the ELC resonator, and (**b**) the proposed IDC resonator.

**Figure 2:**
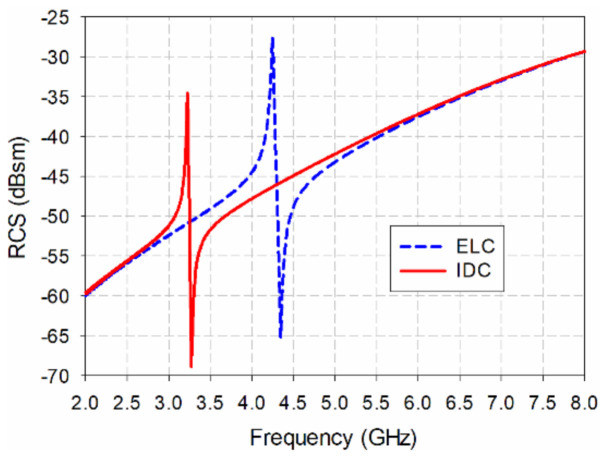
Comparison of the simulated monostatic RCS for the ELC resonator and the proposed IDC resonator.

Figure 1 shows the geometry of the existing ELC and the proposed IDC resonators. The resonators are printed on one side of the substrate, *L* and *W*, respectively. The length and width of the square loop are *l* and *w*, respectively. In designing the resonators, an RF-301 substrate is used with a relative permittivity (*ε*_r_) of 2.97, thickness (*h*) = 0.8 mm, and loss tangent (tan *δ*) = 0.0012. Table 1 shows the final design parameters of the ELC and the proposed IDC resonators. The length of the square loop and the width of the strip of the two resonators are 8 mm and 0.5 mm, respectively. Figure 2 compares the monostatic RCS of the ELC and the proposed IDC resonators. RCS is defined as the ratio of the power of the scattered wave returned from the object to the power of the transmitted electromagnetic wave, and represents the ability of an object to reflect the electromagnetic energy sent [32]. The units for the RCS are expressed in square meters (m^2^) or decibels per square meter (dBsm). When the transmission and reception locations are the same, it is called a monostatic RCS, whereas it is a bistatic RCS when the transmission and reception locations are different.

We see from Figure 2 that the ELC resonator has a resonant peak at 4.245 GHz, and the RCS value is −27.58 dBsm. The proposed IDC resonator has a resonance peak at 3.22 GHz, and the RCS value is −34.55 dBsm. Since the length of the square loop constituting the resonator is the same, the size of the resonator is reduced by 24.2% based on the resonance peak frequency, and the RCS value is reduced by 6.97 dB due to the size reduction.

**Figure 3:**
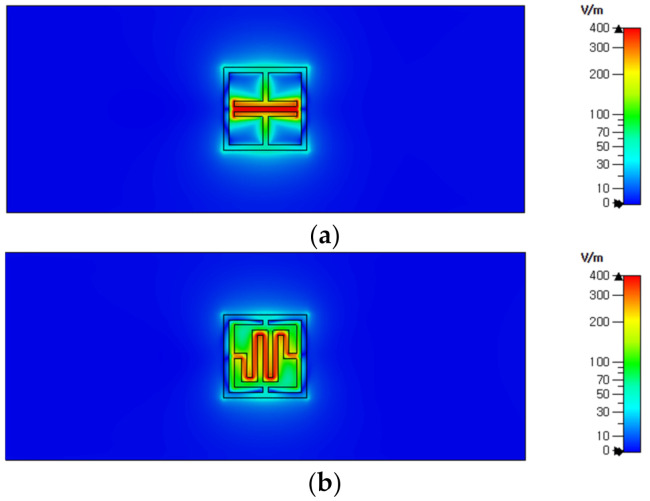
Electric field distributions at resonant peak frequencies for (**a**) the ELC resonator (4.245 GHz), and (**b**) the proposed IDC resonator (3.22 GHz).

Figure 3 compares the electric field distributions at the resonant peak frequency of the ELC resonator and the proposed IDC resonator. In the ELC resonator, the electric field is concentrated between the capacitor-shaped plates, whereas in the proposed IDC resonator, it is concentrated between the plates of the IDC structure. It can be seen that the length of the plate of the IDC structure is longer than that of the conventional capacitor-shaped plate, so the equivalent capacitance of the resonator is larger, and the resonance peak frequency shifts to a lower frequency because of this.

**Figure 4:**
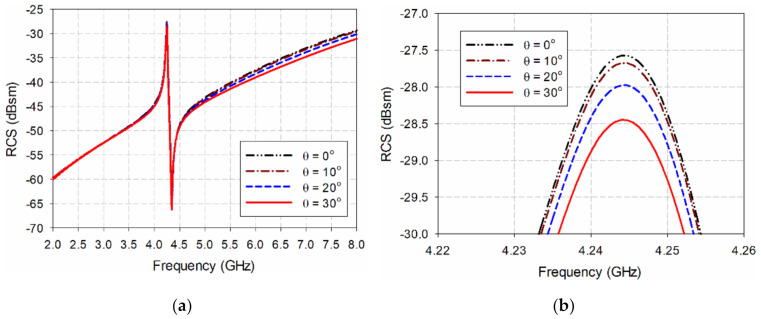
RCS characteristics of the ELC resonator when varying the incident angle: (**a**) 2–8 GHz, (**b**) 4.22–4.26 GHz.

**Figure 5:**
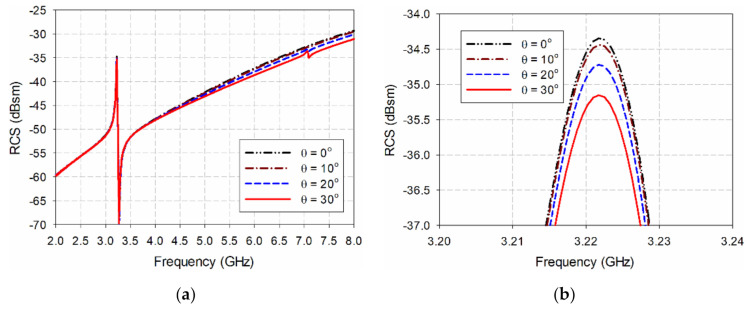
RCS characteristics of the proposed IDC resonator from varying the incident angle: (**a**) 2–8 GHz, (**b**) 3.20–3.24 GHz.

Since wideband horn antennas must be used separately for transmission and reception in actual RCS measurement, a bistatic RCS must be used. Figure 4 and Figure 5 compare the bistatic RCS characteristics according to the incident angle of the transmitting side with the monostatic RCS for the ELC resonator and the proposed IDC resonator. The angle of incidence, θ, is the angle in the z–x plane with respect to the z-axis, and it is a monostatic RCS characteristic when the angle of incidence is 0°. We see that the resonant peak frequencies of the two resonators do not change, and the RCS value at the resonant peak frequency decreases slightly. For the ELC resonator, the resonant peak frequency stays constant at 4.244 GHz, and the RCS value decreases by 0.88 dB, from −27.57 dBsm at a 0° incident angle to −28.45 dBsm at a 30° incident angle. For the proposed IDC resonator, the resonant peak frequency remains constant at 3.222 GHz, and the RCS value decreases by 0.80 dB, from −34.35 dBsm at a 0° incident angle to −35.15 dBsm at a 30° incident angle.

**Figure 6:**
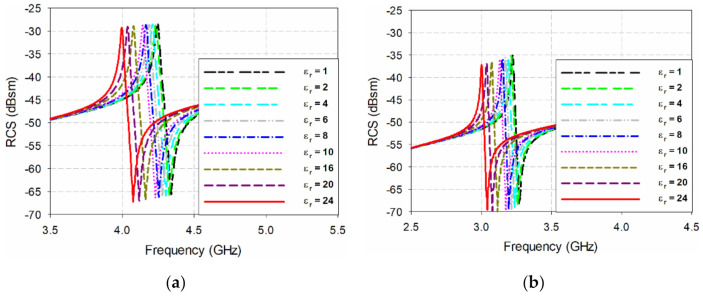
Comparison of RCS characteristics when varying the relative permittivity of the coated dielectric material for (**a**) the ELC resonator, and (**b**) the proposed IDC resonator.

In order to compare the sensitivity of the two resonators when they are coated with a polymer material where the relative permittivity varies according to the humidity, the change in RCS resonance peak frequency according to the relative permittivity change is compared when a 0.02 mm-thick dielectric material is coated on the surface of the two resonators, as shown in Figure 6. When the relative permittivity of the coated dielectric material was 6, the resonant peak frequency of the ELC resonator decreased by 1.41% to 4.185 GHz, and the bistatic RCS value decreased by 0.17 dB to −28.63 dBsm. In the proposed IDC resonator, the resonant peak frequency was reduced by 1.55% to 3.17 GHz, and the bistatic RCS value was reduced by 0.37 dB to −35.70 dBsm. When the relative permittivity of the coated dielectric material increased to 24, the resonance peak frequency of the ELC resonator decreased by 5.89% to 3.995 GHz, and the bistatic RCS value decreased by 0.78 dB to −29.24 dBsm. In the proposed IDC resonator, the resonant peak frequency dropped by 6.83% to 3 GHz, and the bistatic RCS value dropped by 1.98 dB to –37.31 dBsm. Therefore, we can see that the proposed IDC resonator has a larger variation in both RCS resonance peak frequency and RCS value with respect to the relative permittivity change, compared to the conventional ELC resonator, and that the sensitivity improved.

**Figure 7:**
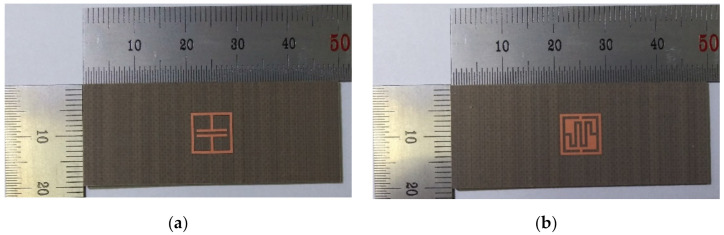
Photograph of the fabricated resonators: (**a**) the ELC resonator, and (**b**) the proposed IDC resonator.

**Figure 8:**
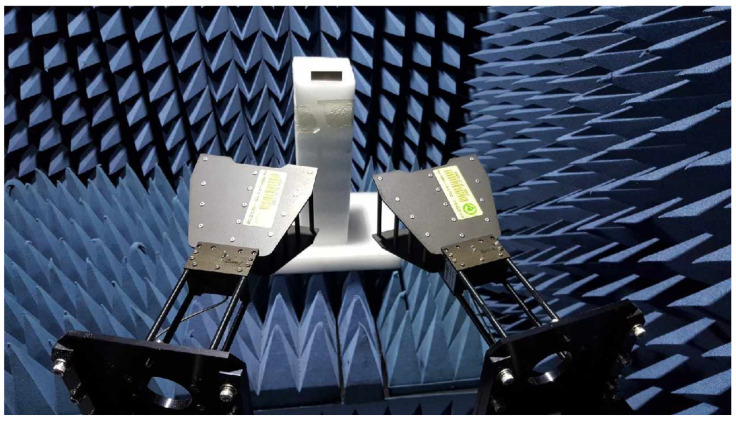
Measurement setup in an anechoic chamber.

For RCS performance validation, the ELC resonator and the proposed IDC resonator were fabricated using an RF-301 substrate, as shown in Figure 7. Figure 8 shows the measurement setup in an anechoic chamber for RCS measurement. An N5230A vector network analyzer (Agilent, Santa Rosa, CA, USA) was used to measure the transmission coefficient (S_21_) using the transmit and receive antennas, and double-ridged horn antennas (C&G Microwave co. ltd., Daejeon, Korea) covering 2–18 GHz were used as the transmitting and receiving antennas. The distance between the transmitting/receiving antennas and the resonator under test was about 300 mm, and the transmit antenna had an incident angle of about 30° from the vertical direction of the tag surface.

The measured bistatic RCS value $\sigma_{tag}$ of the two resonators was calculated using the following formula [32]:

(1) $$\sigma_{tag}={\left[\frac{S_{21,\;tag}-S_{21,\;air}}{S_{21,\;ref}-S_{21,\;air}}\right]}^{2}\sigma_{ref}$$

where $\sigma_{ref}$ is the RCS value of the object to be used as a reference, $S_{21,\;tag}$ is the measured transmission coefficient when the resonator is placed, $S_{21,\;ref}$ is the measured transmission coefficient when the reference object is placed, and $S_{21,\;air}$ is the transmission coefficient in air measured in the absence of an object. A copper wire with a length of 30 mm and a diameter of 0.4 mm was used as a reference object.

**Figure 9:**
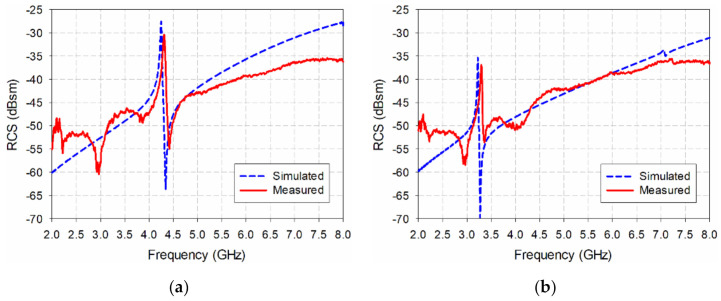
Measured bistatic RCS characteristics of (**a**) the ELC resonator, and (**b**) the proposed IDC resonator.

Figure 9 compares the simulated results with the measured bistatic RCS values for the ELC resonator and the proposed IDC resonator. In the ELC resonator, the resonant peak frequency and the value of the simulated bistatic RCS were 4.245 GHz and −28.46 dBsm respectively. The measured resonant peak frequency of the bistatic RCS increased by 1.41% to 4.305 GHz, while the measured RCS value decreased by 1.93 dB to −30.39 dBsm. For the proposed IDC resonator, the resonant peak frequency and the value of the simulated bistatic RCS were 3.22 GHz and −35.33 dBsm respectively, while the measured resonant peak frequency of the bistatic RCS increased by 2.33% to 3.295 GHz, and the measured RCS value decreased by 1.58 dB to −36.91 dBsm. The difference between the measured and simulated results might be caused by measurement setup and/or a manufacturing error in the resonator.

## 3. Design of the Eight-Bit Chipless RFID Tag

**Figure 10:**
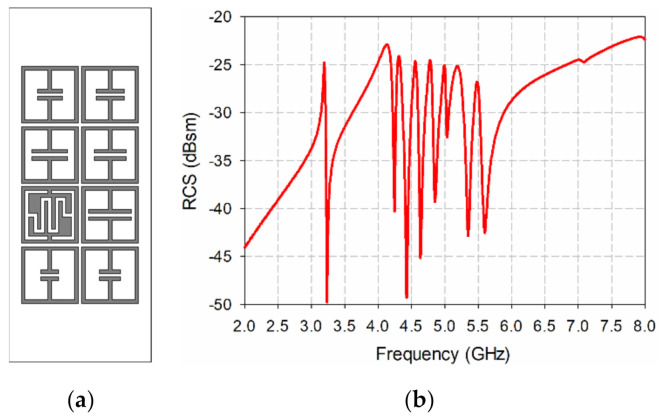
The eight-bit chipless RFID tag: (**a**) the geometry, and (**b**) the simulated bistatic RCS.

An eight-bit chipless RFID tag with a two-by-four array was designed as shown in Figure 10a. It consists of one IDC-based resonator and seven ELC resonators to provide humidity and identification information simultaneously. For humidity sensing, the IDC resonator with an unloaded resonant peak frequency at 3.195 GHz is used, whereas the seven ELC resonators are designed to resonate at 4.135 GHz (*l*_1_ = 6 mm), 4.31 GHz (*l*_1_ = 5 mm), 4.56 GHz (*l*_1_ = 4.5 mm), 4.78 GHz (*l*_1_ = 4 mm), 4.995 GHz (*l*_1_ = 3.5 mm), 5.19 GHz (*l*_1_ = 3 mm), and 5.485 GHz (*l*_1_ = 2.5 mm), as shown in Figure 10b, by means of adjusting the length of capacitor-shaped plate. The IDC resonator is located second from the left at the bottom of the two-by-four arrangement on a 20 mm × 50 mm RF-301 substrate. The length of the square loop and the width of the strip of the IDC and ELC resonators are 8 mm and 0.5 mm, respectively. The spacing between the resonators is 0.5 mm.

**Figure 11:**
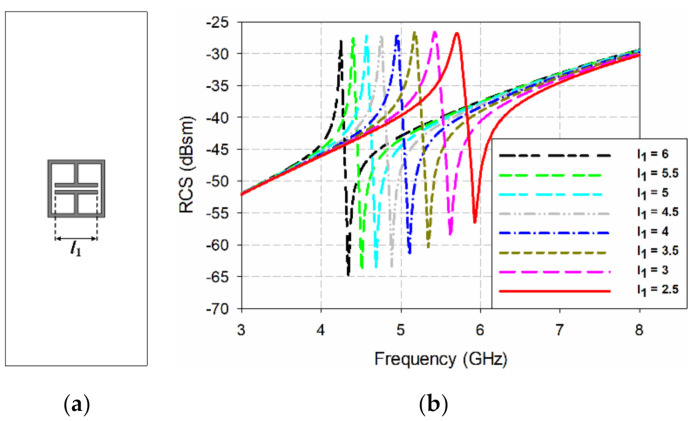
Bistatic RCS characteristics of the ELC resonator from varying the length of the capacitor-shaped plate: (**a**) the geometry, and (**b**) the simulated bistatic RCS.

Figure 11 shows the effects on the bistatic RCS characteristics of varying the length of capacitor-shaped plate *l*_1_ in the ELC resonator. The length of capacitor-shaped plate *l*_1_ was varied between 6 mm and 2.5 mm, with other parameters fixed as seen in Table 1. When *l*_1_ = 6 mm, the ELC resonator had a resonant peak at 4.244 GHz and the RCS value was −28.03 dBsm, whereas the resonant peak moved towards a higher frequency at 5.703 GHz with an RCS value of −26.81 dBsm when *l*_1_ was decreased to 2.5 mm. Therefore, we can change the position of the resonant peak for the ELC resonator by varying the length of capacitor-shaped plate *l*_1_.

**Figure 12:**
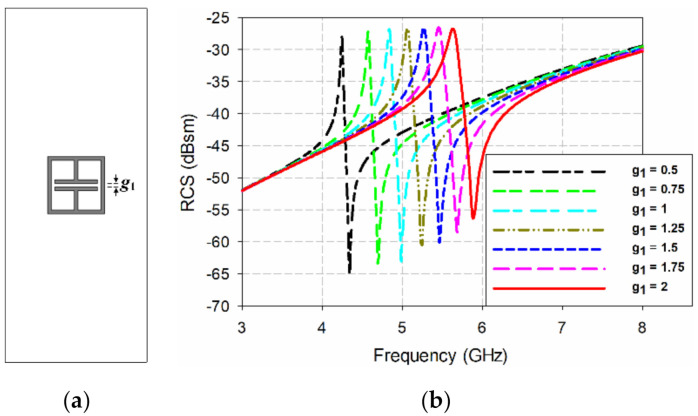
Bistatic RCS characteristics of the ELC resonator from varying the gap between the capacitor-shaped plates: (**a**) the geometry, and (**b**) the simulated bistatic RCS.

Another way to change the position of the resonant peak for the ELC resonator is by varying the gap between the capacitor-shaped plates (*g*_1_). Figure 12 shows the effects on the bistatic RCS characteristics of varying the gap *g*_1_ between the capacitor-shaped plates. The gap *g*_1_ between the capacitor-shaped plates was varied between 0.5 mm and 2 mm, while the other parameters remained fixed (see Table 1). When *g*_1_ = 0.5 mm, the ELC resonator had a resonant peak at 4.244 GHz, and the RCS value was −28.03 dBsm, whereas the resonant peak moved towards a higher frequency at 5.632 GHz with an RCS value of −26.77 dBsm when *g*_1_ was increased to 2 mm.

**Figure 13:**
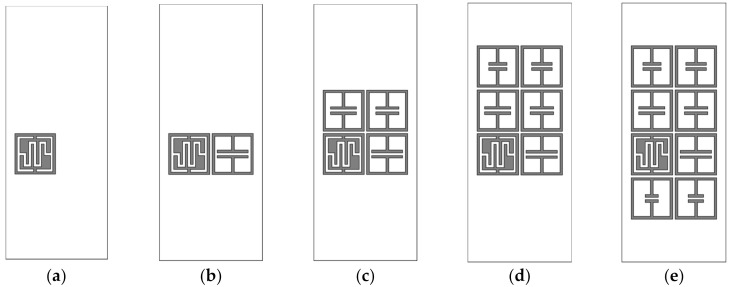
Geometries of the array configurations for RCS comparison: (**a**) the single IDC resonator, (**b**) the two-by-one configuration, (**c**) the two-by-two configuration, (**d**) the two-by-three configuration, (**e**) the two-by-four configuration.

**Figure 14:**
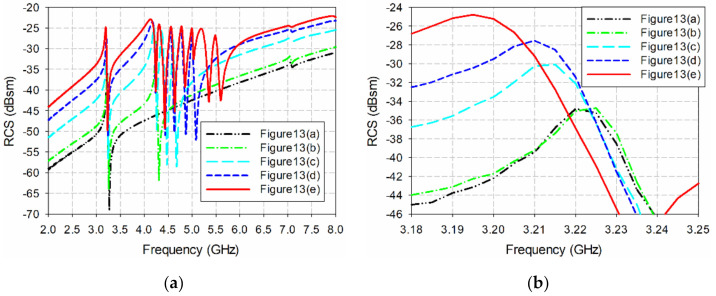
RCS comparison for the five configurations shown in Figure 13: (**a**) 2–8 GHz, and (**b**) 3.18–3.25 GHz for the IDC resonator.

Next, we investigated the effects of varying the number of elements and array configuration on the resonant peak frequency and RCS magnitude of the IDC resonator. The RCS characteristics of the two-by-four configuration is compared with a single IDC resonator, a two-by-one configuration with an IDC resonator and an ELC resonator, a two-by-two configuration with an IDC resonator and three ELC resonators, and a two-by-three configuration with an IDC resonator and five ELC resonators. The geometries of the five configurations are shown in Figure 13, and their RCS characteristics are plotted in Figure 14.

The single IDC resonator in Figure 13a had a resonant peak at 3.22 GHz with an RCS value of −34.80 dBsm. For the two-by-one configuration with the IDC resonator and an ELC resonator with *l*_1_ = 6 mm in Figure 13b, the resonant peak of the IDC resonator appeared at 3.225 GHz with an RCS value of −34.72 dBsm, and these results are similar to those of the single IDC resonator. When two ELC resonators with *l*_1_ = 5 mm and 4.5 mm were appended to make a two-by-two configuration in Figure 13c, the frequency of the resonant peak for the IDC resonator shifted towards a lower frequency at 3.215 GHz with an RCS value of −30.08 dBsm. In this case, the resonant peak shift is about 0.005 GHz (0.16%), and the increase in the RCS is about 4.72 dB (13.56%), compared to the single IDC resonator. When two more ELC resonators with *l*_1_ = 4 mm and 3.5 mm were appended to make a two-by-three configuration (see Figure 13d), the frequency of the resonant peak for the IDC resonator shifted towards a lower frequency at 3.21 GHz with an RCS value of −27.55 dBsm. The resonant peak shift is about 0.01 GHz (0.31%) and the increase in the RCS is about 7.25 dB (20.83%) compared to the single IDC resonator.

Finally, when two more ELC resonators with *l*_1_ = 3 mm and 2.5 mm were appended to make a two-by-four configuration in Figure 13e, the frequency of the resonant peak for the IDC resonator shifted towards a lower frequency at 3.195 GHz with RCS value of −24.82 dBsm. The resonant peak shift is about 0.025 GHz (0.78%), and the RCS increase is about 9.98 dB (28.68%), compared to the single IDC resonator. The results for the resonant peak frequencies and RCS values of the IDC resonator in the five configurations are summarized in Table 2. We see that the RCS value of the resonant peak frequency for the IDC resonator increases as the number of array elements placed nearby increases due to the mutual coupling among the elements. The increase in the RCS value becomes larger as the number of arrays increases in the vertical direction. On the other hand, the variation in the resonant peak frequency is relatively small (less than 0.78%).

## 4. Humidity Sensing Experiment Result and Discussion

**Figure 15:**
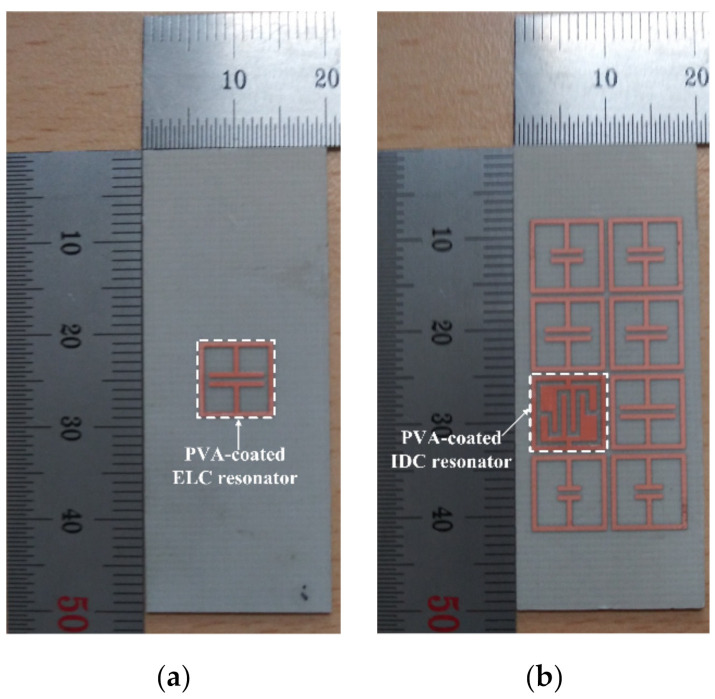
The fabricated RFID tags: (**a**) a single ELC resonator, and (**b**) the proposed eight-bit chipless RFID tag in a two-by-four array.

In order to validate the performance of the proposed eight-bit chipless RFID tag in a two-by-four array, it was fabricated on a 20 mm × 50 mm RF-301 substrate. A single ELC resonator was also fabricated to compare against the humidity sensing performance of the IDC resonator. Figure 15 shows the fabricated single ELC resonator and the proposed eight-bit chipless RFID tag. 

A hygroscopic polymer material, PVA, coated the single ELC resonator and the IDC-based resonator in a two-by-four array at a thickness of 0.02 mm for the comparison of humidity sensing performance. The PVA (degree of polymerization = 1,500, degree of saponification = 99 mol%) was purchased from Yakuri Pure Chemicals Co., Ltd (Kyoto, Japan) [30]. The PVA solution (a 5 wt% concentration) was prepared by dissolving the PVA in deionized water. The surface areas on the single ELC resonator and the IDC resonator of the proposed eight-bit chipless RFID tag were coated by brushing on 14 mg of the PVA solution, and the PVA-coated resonators were then dried in a convection oven for 120 min at 60 °C. 

**Figure 16:**
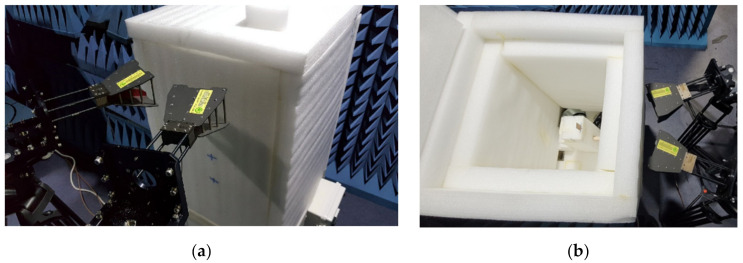
(**a**) The measurement setup with a non-reflective temperature and humidity chamber, and (**b**) the tag inside the temperature and humidity chamber.

In order to measure the bistatic RCS change in the single ELC resonator and the IDC resonator in the proposed eight-bit chipless RFID tag based on the relative humidity (RH), a non-reflective temperature and humidity chamber was manufactured using Styrofoam, as shown in Figure 16. The outer dimension of the Styrofoam chamber is 500 mm (L) × 510 mm (W) × 830 mm (H). The thickness of Styrofoam is 10 mm in all directions. The temperature and humidity control units of the chamber is placed below the Styrofoam chamber. A humidifier was used to increase the RH, whereas two fans were used to decrease the RH. The tag under test was placed in the middle of the chamber, as shown in Figure 16b. The RH was varied from 50% to 80% in 10% intervals at a temperature of 25 °C in order to measure the bistatic RCS of the proposed tag. For each RH, transmission coefficient S_21_ was measured after waiting five minutes to stabilize the humidity inside the chamber. It was measured using an N5230A vector network analyzer (Agilent) and two double-ridged horn antennas (C&G Microwave). The non-reflective temperature and humidity chamber was placed in front of the two antennas in an anechoic chamber. The distance between the transmitting/receiving antennas and the resonator under test was also kept at about 300 mm, and the same incident angle of about 30° from the vertical direction of the tag surface was applied to the transmitting antenna.

**Figure 17:**
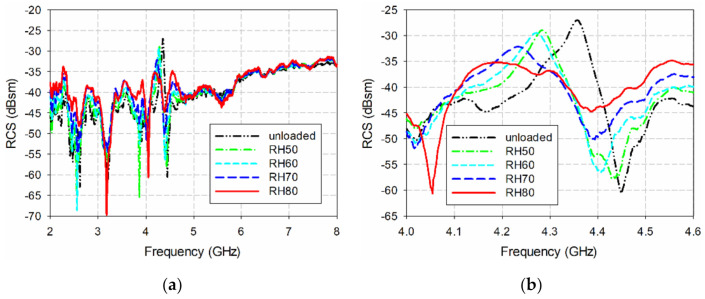
Measured bistatic RCS characteristics of the single ELC resonator from varying the RH: (**a**) 2–8 GHz, (**b**) 4.0–4.6 GHz.

Figure 17 shows the measured bistatic RCS characteristics of the single ELC resonator from varying the RH from 50% to 80%. For unloaded conditions, the measured resonant peak of the ELC resonator occurred at 4.356 GHz with an RCS value of −27.03 dBsm. Note that the simulated resonant peak of the ELC resonator appeared at 4.244 GHz with an RCS value of −28.03 dBsm, as shown in Figure 11b. When RH was 50%, the measured resonant peak increased to 4.284 GHz with an RCS value of −28.96 dBsm. When the RH increased to 80%, the measured resonant peak increased to 4.186 GHz with an RCS value of −35.17 dBsm. Measured resonant peak frequencies and RCS values of the single ELC resonator along with those of the IDC resonator in the proposed eight-bit chipless RFID tag are summarized in Table 3 and Table 4.

**Figure 18:**
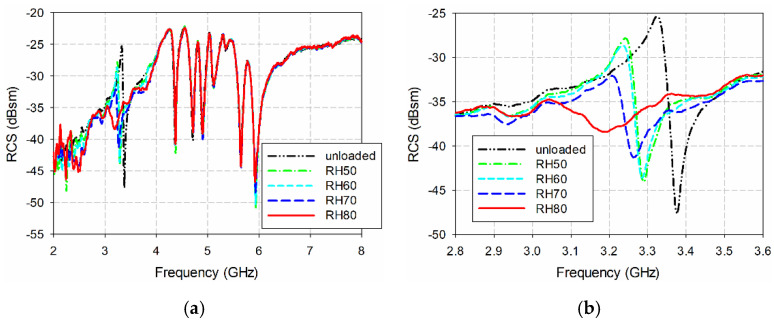
Measured bistatic RCS characteristics of the IDC resonator in the proposed eight-bit chipless RFID tag for varying RH levels: (**a**) 2–8 GHz, (**b**) 2.8–3.6 GHz.

Figure 18 shows the measured bistatic RCS characteristics of the proposed eight-bit chipless RFID tag from varying the RH between 50% and 80%. For unloaded conditions, the measured resonant peak of the IDC resonator occurred at 3.324 GHz with an RCS value of −25.30 dBsm. We note that the simulated resonant peak of the IDC resonator in the eight-bit chipless RFID tag appeared at 3.195 GHz with an RCS value of −24.82 dBsm, as shown in Figure 14. When the RH was 50%, the measured resonant peak increased to 3.240 GHz with an RCS value of −27.83 dBsm. When the RH further increased to 80%, the measured resonant peak increased to 3.044 GHz with an RCS value of −34.74 dBsm. It is also noticeable that the resonant peak frequencies and RCS values of the other seven ELC resonators in the proposed eight-bit chipless RFID tags without PVA coating are almost constant for the varying RH levels, as shown in Figure 18a.

**Figure 19:**
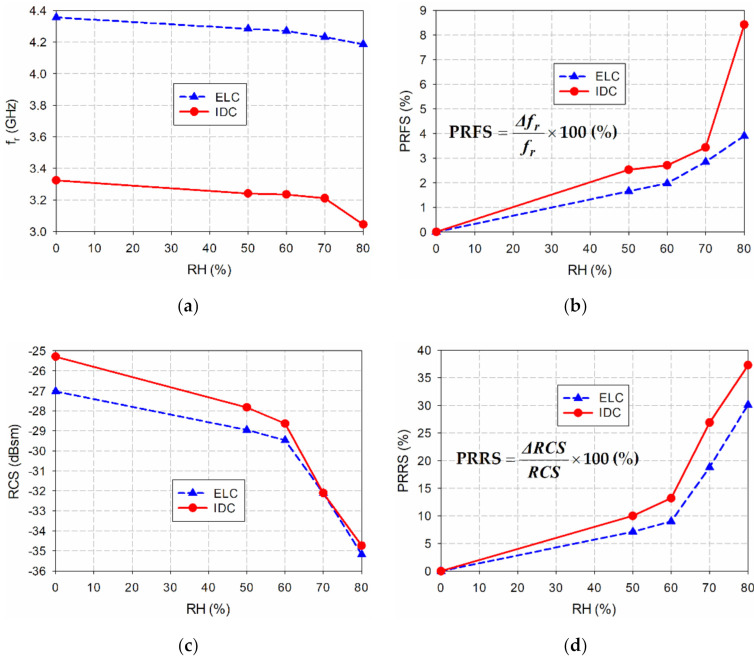
Performance comparison of the single ELC resonator and the IDC resonator in the proposed eight-bit chipless RFID tag for varying RH: (**a**) *f*_r_; (**b**) PRFS; (**c**) RCS value; (**d**) PRRS.

Next, the measured resonant peak frequencies, the percent relative frequency shifts (PRFSs), the RCS values, and the percent relative RCS shifts (PRRSs) of the single ELC resonator and the IDC resonator in the proposed eight-bit chipless RFID tag are compared in Figure 19 as a function of RH. We note that both PRFSs and PRRSs are calculated based on the resonant peak frequency and the RCS value for unloaded conditions (RH = 0%). The definitions of PRFS and PRRS are shown as insets in Figure 19b,d.

For the single ELC resonator, the PRFS increased from 1.65% to 3.90% when RH increased from 50% to 80%, whereas it increased from 2.53% to 8.4% for the IDC resonator in the proposed eight-bit chipless RFID tag. The PRRS increased from 7.12% to 30.10% for the single ELC resonator when RH increased from 50% to 80%, whereas it increased from 10% to 37.3% for the IDC resonator in the proposed eight-bit chipless RFID tag. Therefore, we can see that the sensitivities in both the resonant peak frequency and the RCS value of the IDC resonator are better than those of the ELC resonator. Furthermore, the variation in PRRS is much larger, compared to the PRFS.

The sensitivity based on the resonant frequency shift in MHz/RH is compared for the single ELC resonator and the IDC resonator in the proposed eight-bit chipless RFID tag. When RH varied from 50% to 60%, the sensitivity was 1.4 MHz/RH for the single ELC resonator, whereas it was 0.6 MHz/RH for the IDC resonator in the proposed eight-bit chipless RFID tag. When RH varied from 60% to 70%, the sensitivity was 3.8 MHz/RH for the single ELC resonator, whereas it was 2.4 MHz/RH for the IDC resonator in the proposed eight-bit chipless RFID tag. When RH varied from 70% to 80%, the sensitivity was 4.6 MHz/RH for the single ELC resonator, whereas it was 16.6 MHz/RH for the IDC resonator in the proposed eight-bit chipless RFID tag. Note that although the sensitivity value of the single ELC resonator is larger than that of the IDC resonator in the proposed eight-bit chipless RFID tag in the range from 50% to 70%, the relative sensitivity considering the resonant frequency of the IDC resonator in the proposed eight-bit chipless RFID tag is larger than that of the single ELC resonator. When the sensitivity in MHz/RH was calculated in the full RH range from 50% to 80%, the sensitivity was 3.27 MHz/RH for the single ELC resonator, whereas it was 6.53 MHz/RH for the IDC resonator in the proposed eight-bit chipless RFID tag.

Table 5 compares the humidity sensing performance of the IDC resonator in the proposed eight-bit chipless RFID tag with other humidity sensing chipless RFID tags in the literature. We note that sensing material, resonator type, coating thickness, and humidity exposure time are all different. The thicker the coating and the longer the humidity exposure time, the shifts in the resonant frequency and the RCS value would be larger. We can see that the size of the proposed IDC resonator in terms of the free space wavelength of the resonator (*l*/*λ*_0_) is the smallest among the resonators in Table 5. In addition, both sensitivities in MHz/RH and Δf_r_/f_r_ of the proposed IDC resonator are the highest among them. Note that the sensitivities are calculated in the RH range specified in the literature. 

## 5. Conclusions

The method for designing an eight-bit chipless RFID tag with humidity-sensing ability and identification information was studied in this paper. First, a compact resonator was designed by employing the IDC structure instead of the capacitor-shaped structure in the ELC resonator in order to enhance the permittivity sensitivity when varying the RH. Next, an eight-bit chipless RFID tag was designed in a two-by-four configuration, consisting of one IDC-based resonator for humidity sensing and seven ELC resonators for identification information. It was found that the RCS value of the IDC resonator in the proposed eight-bit chipless RFID tag increased considerably, compared to the single IDC resonator, due to the mutual coupling effect among the array elements.

We confirmed by experiment that the sensitivities in both resonant peak frequency and RCS value of the IDC resonator in the proposed eight-bit chipless RFID tag were better than the ELC resonator when RH increased from 50% to 80%. For both IDC and ELC resonators, the RCS value varied much more, compared to the resonant peak frequency. The resonant peak frequencies and RCS values of the seven resonators without a PVA coating in the eight-bit chipless RFID tag did not change when varying the RH.

The proposed chipless RFID tag is expected to be used for wireless sensing of ambient humidity in various IoT applications. As future work, we will try to study a polymer material where the relative permittivity changes according to the temperature, and we will use it to develop a chipless RFID tag that can measure temperature and humidity simultaneously.

## Figures and Tables

**Table 1:** Design parameters of the ELC resonator and the proposed IDC resonator.

Parameter	Value (mm)	Parameter	Value (mm)
*L*	50	*g*_3_	0.5
*W*	20	*l*_2_	6
*l*	8	*g*_4_	0.5
*w*	0.5	*g*_5_	0.5
*l*_1_	6	*g*_6_	0.5
*g*_1_	0.5	*h*	0.8
*g*_2_	2.75		

**Table 2:** Comparison of the resonant peak frequencies and the RCS values for the IDC resonator in the five configurations.

Array Configuration	f_r_ (GHz)	Δf_r_ (GHz)	RCS (dBsm)	ΔRCS (dB)
Single IDC	3.220	0	−34.80	0
2-by-1	3.225	+0.005 (+0.16%)	−34.72	+0.08 (+0.23%)
2-by-2	3.215	−0.005 (−0.16%)	−30.08	+4.72 (+13.56%)
2-by-3	3.210	−0.01 (−0.31%)	−27.55	+7.25 (+20.83%)
2-by-4	3.195	−0.025 (−0.78%)	−24.82	+9.98 (+28.68%)

**Table 3:** Resonant peak frequencies (in gigahertz) at various RH levels for the single ELC resonator and the IDC resonator in the proposed eight-bit chipless RFID tag coated with PVA.

Resonators	Unloaded	RH 50%	RH 60%	RH 70%	RH 80%
ELC	4.356	4.284	4.270	4.232	4.186
IDC	3.324	3.240	3.234	3.210	3.044

**Table 4:** Measured RCS values (in dBsm) at various RH levels for the single ELC resonator and the IDC resonator in the proposed eight-bit chipless RFID tag coated with PVA.

Resonators	Unloaded	RH 50%	RH 60%	RH 70%	RH 80%
ELC	−27.03	−28.96	−29.46	−32.11	−35.17
IDC	−25.30	−27.83	−28.64	−32.11	−34.74

**Table 5:** Humidity sensing performance comparison with other humidity-sensing chipless RFID tags in the literature.

	Resonator Type	Resonator Size (*l*/*λ*_0_)	Coating Material	Coating Thickness (mm)	f_r_ (GHz)	RH Range (%)	MHz/RH	Δf_r_/f_r_ (%)
[25]	Coupled-loop resonators	0.377	SiNW	0.017	3.33	74–98	1.46	1.05
[26]	IDC	0.0002	GO	-	0.0352	50–95	0.02	2.07
[28]	ELC	0.139	PVA	0.1	6.96	35–85	5.40	3.96
This Work	ELC	0.116	PVA	0.02	4.356	50–80	3.27	2.29
	IDC	0.089	PVA	0.02	3.324	50–80	6.53	6.05

## Data Availability

Not applicable.
